# P-2268. *Candida tropicalis* Invasive Infection in Febrile Neutropenia, a High Mortality Infection

**DOI:** 10.1093/ofid/ofae631.2421

**Published:** 2025-01-29

**Authors:** Marcos Arango, Isabel C Ramirez

**Affiliations:** Hospital Pablo Tobon Uribe, medellin, Antioquia, Colombia; Hospital Pablo Tobon Uribe, Universidad de Antioquia, Medellin, Antioquia, Colombia

## Abstract

**Background:**

Invasive candidiasis due to species other than *Candida albicans* emerges as a problem in neutropenic patients receiving antifungal prophylaxis, due to decreased susceptibility to triazoles. *Candida tropicalis* is the most frequently species in this population and is associated with higher mortality when compared *to Candida albicans*. The aim of this study is to characterize the neutropenic population with invasive candidiasis and establish differences in outcome of patients with *Candida tropicalis* infection compared to other *Candida* species.

Characteristics of patients with invasive candidiasis

**Figure:**
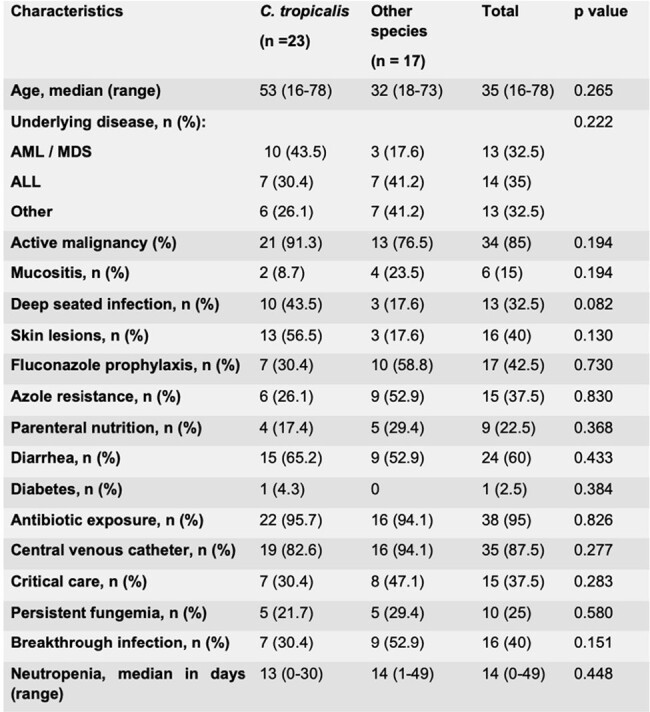

**Methods:**

Retrospective cohort study of patients with hematological malignancies undergoing chemotherapy who developed invasive candidiasis between 2010 and 2022. Comparison of two qualitative variables was performed using Pearson's Chi-square test or Fisher's exact test. Estimation of group survival was performed using the Kaplan-Meier method and comparison of survival functions with the Log-Rank test.

Results of multivariate analysis of risk factors associated with adverse outcomes.

**Figure:**
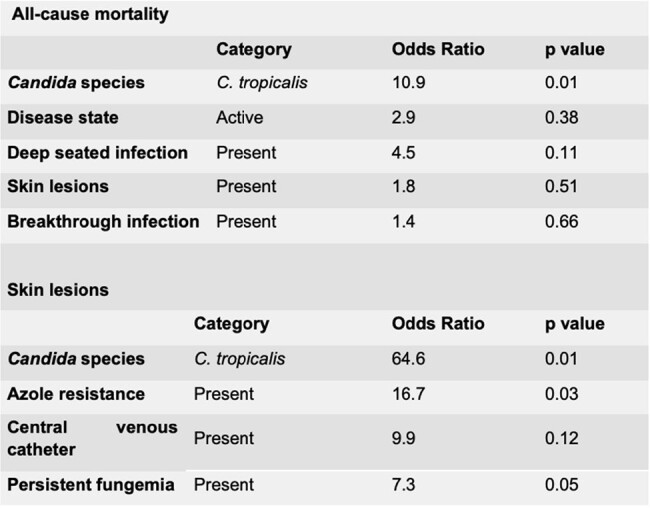

**Results:**

At the time of fungemia, 85% of patients had active malignancy, with a median of 14 days of neutropenia until the development of infection. 15% of patients had mucositis and up to 60% had concomitant diarrhea. 95% had previously received antibiotics and 87.5% had a central catheter. In 40% of the patients, the infection occurred as a breakthrough infection and in 37.5% it was associated with azole resistance. 32.5% of cases were associated with deep-seated infection and 25% had persistent fungemia. The 30-day mortality of patients with candidemia due to *C. tropicalis* was higher than that experienced by subjects with candidemia due to other species (69% vs. 35%, p = 0.034). The median overall survival was 15 days (95% CI 12-18) for the first group. Multivariate analysis showed that *C. tropicalis* infection was associated with an increased risk of all-cause mortality. The risk factors associated with cutaneous lesions were *C. tropicalis* infection and azul resistance.

Overall survival of study patients

**Figure:**
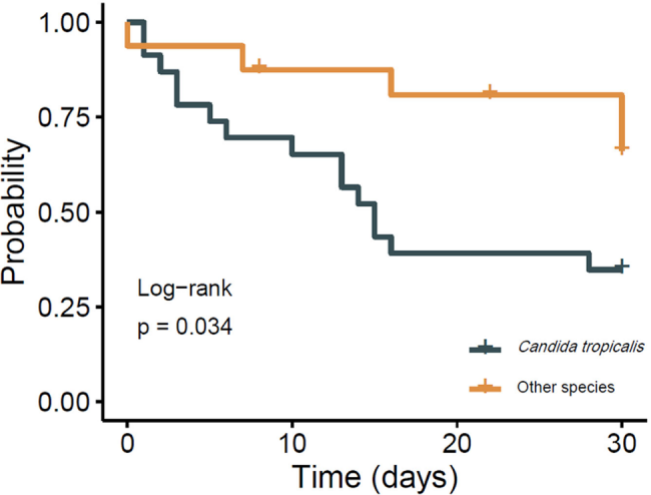

**Conclusion:**

Patients who develop *C. tropicalis* candidemia during febrile neutropenia after chemotherapy for hematologic malignancies have a higher mortality than those infected with other *Candida* species. Azole resistance emerges as a threat given routine prophylaxis in this population, so mitigation strategies are necessary.

**Disclosures:**

All Authors: No reported disclosures

